# The therapeutic efficacy of 5-ALA based photodynamic therapy and chemotherapy combination in triple negative breast cancer cells

**DOI:** 10.1007/s10103-024-04141-9

**Published:** 2024-07-23

**Authors:** Beyzanur Erk, Ali Furkan Kamanli, Gamze Guney Eskiler

**Affiliations:** 1 Department of Biomedical Engineering, Faculty of Engineering and Natural Sciences, Istinye University, Istanbul, Turkey; 2 Department of Electric and Electronics Engineering, Faculty of Technology, Sakarya University of Applied Sciences, Sakarya, Turkey; 3 Department of Medical Biology, Faculty of Medicine, Sakarya University, Korucuk Campus, 54290 Sakarya, Turkey

**Keywords:** Triple negative breast cancer, Photodynamic therapy, 5-ALA, Cisplatin, Apoptosis

## Abstract

Triple negative breast cancer (TNBC) is one of the subtypes of breast cancer characterized by a heterogeneous and aggressive nature. Photodynamic therapy (PDT) has drawn significant attention in cancer treatment. However, solubility of photosensitizer, penetration problems into a target tissue and insufficient oxygen concentration limit the effectiveness of PDT. To overcome these limitations and to reduce the side effects of chemotherapy, combination treatment modalities play an essential role in cancer treatment. In this study, we aimed to investigate the combination efficacy of cisplatin-based chemotherapy and 5-Aminolevulinic acid (5-ALA)/PDT in TNBC cells and healthy breast cells in vitro. To determine the effect of the combination effects of cisplatin and 5-ALA/PDT on TNBC cells, two treatment protocols (simultaneous and sequential combination therapy) were evaluated compared with cisplatin and 5-ALA/PDT monotherapy and WST-1, Annexin V assay, acridine orange (AO) and mitochondrial staining were performed. Our findings showed that MDA-MB-231 TNBC cell viability was significantly decreased following simultaneous combination treatment compared to cisplatin and 5-ALA/PDT monotherapy. Additionally, simultaneous combination treatment was more effective than sequential combination treatment. The simultaneous combination treatment of 2.5 µM cisplatin and 5-ALA/PDT at 6 J/cm^2^ and 9 J/cm^2^ induced 46.78% and 53.6% total apoptotic death, respectively in TNBC cells compared with monotherapies (cisplatin (37.88%) and 5-ALA/PDT (6 J/cm^2^: 31.48% and 9 J/cm^2^: 37.78%). Additionally, cisplatin and 5-ALA/PDT combination treatment resulted in nuclear fragmentation and mitochondrial damage due to apoptosis. Our results suggest that cisplatin and 5-ALA/PDT simultaneous combination therapy could be a promising new alternative strategy for treating TNBC. However, further studies are required to assess the underlying molecular mechanisms of cisplatin and 5-ALA/PDT combination treatment at the molecular level.

## Introduction

Triple negative breast cancer (TNBC) is a subtype of breast cancer and constitutes approximately 10–20% of breast cancer diagnoses. TNBC exhibits a unique molecular profile due to a lack of the estrogen receptor (ER), progesterone receptor (PR), and human epidermal growth factor receptor 2 (HER2) expression with distant metastasis potential, aggressive behavior, short overall survival, and lower 5-year relative survival rate compared to other breast cancer subtypes [1–3]. In addition, TNBC tumors usually have aggressive features such as more advanced stage, larger tumor size, and lymph node positivity and their incidence varies according to age, ethnicity, socioeconomic status, and survival rates [4]. Although the primary option in the treatment of TNBC is chemotherapy, there are also treatment options such as surgery, radiotherapy, and immunotherapy. However, due to more aggressive features and poor prognosis than other breast cancer subtypes, specific effective treatment options for TNBC patients need to be developed [5].

Photodynamic therapy (PDT) is based on photosensitive substances excited by light of a specific wavelength and, interacting with molecular oxygen in the target tissue and causing cell death in a specific way. PDT exhibits low side effects as a non-invasive method, and thus, it can be used in different medical fields, including dermatology, gynecology, urology, oncology, etc. [6–8]. PDT consists of three main components: photosensitizer (PS), light, and oxygen. After the PS absorbs light of a particular wavelength, it is activated and transferred to the excited form. The stimulated form of PS interacts with the oxygen molecule in the target cell and produces reactive oxygen species (ROS), thus causing damage to the target cell [9]. In this context, PDT exerts anticancer effects through three mechanisms: (i) directly causing death in cancer cells due to ROS production, (ii) causing vascular damage, and (iii) triggering the activation of the immune system by inducing an inflammatory and immune response.

5-Aminolevulinic acid (5-ALA) is generally selected for PDT in different types of cancer [10, 11]. In our previous studies, 5-ALA monotherapy induces a considerable decrease in the viability of MCF-7 and MDA-MB-231 breast cancer cells and hepatocellular carcinoma cells upon irradiation with different doses [12, 13]. However, the efficacy of 5-ALA following irradiation can be limited in cancer cells due to disadvantageous features, including oral use and phototoxicity [14]. Furthermore, PS used in PDT has limitations such as insolubility, lower penetration into a target tissue, toxicity in normal cells and inability to obtain sufficient oxygen concentration. At this point, combination therapy can be used to overcome these limitations of PDT [15, 16].

In this context, we assessed the combination effects of cisplatin-based chemotherapy and 5-ALA-based PDT through two different treatment protocols (sequential or simultaneous) following irradiation on TNBC cells.

## Materials and methods

### Cell culture

In this study, triple-negative breast cancer cell line MDA-MB-231 (ATCC, USA, HTB-26) and healthy breast epithelial cell line MCF-10A (ATCC, ABD, CRL-10317) were used to determine the combined effect of 5-ALA-based PDT and cisplatin-based chemotherapy. MDA-MB-231 cells were cultured in DMEM (Gibco, Thermo Fisher Scientific, USA) containing 10% fetal bovine serum (FBS) (Gibco, Thermo Fisher Scientific, USA), 2 mM L-glutamine (Gibco, Thermo Fisher Scientific, USA), 1% penicillin, and streptomycin (Gibco, Thermo Fisher Scientific, USA) and MCF-10A cells were maintained in DMEM F-12 (Gibco, Thermo Fisher Scientific, USA) containing 1 mg/ml hydrocortisone (Sigma Aldrich, USA), 100 mg/ml epidermal growth factor (EGF), 10 mg/ml insulin, 10% FBS, 1% penicillin and streptomycin.

### Photodynamic treatment

The wavelength of the laser light source was 635 nm with ± 3 full-width half maximum (FWHM). The laser light source was used in various pulse frequencies from DC to 20 MHz. In the experiment, continuous wave (CW) mode was performed for irradiation at 1.5 J/cm^2^, 3 J/cm^2^, 6 J/cm^2^, 9 J/cm^2^ and 12 J/cm^2^. The optic power and wavelength spectrum were validated by a power meter and spectrometer (PM100 and C series spectrometer,Thorlabs, Germany). The fluence rate was 30 mW/cm^2^ to irradiate with different light doses.

### Treatment protocols and cell viability

The cells (2 × 10^4^ cells/well) were seeded in 96 well plates and incubated for 24 h at 37 °C with %5 CO_2_. 100 mg of cisplatin (Sigma Aldrich, USA) stock was dissolved in 1 mL of dimethyl sulfoxide (DMSO) and diluted with the medium. After incubation, the cells were treated with cisplatin alone at 1, 2.5, and 5 µM concentrations and incubated for 24 h.

For the PDT protocol, the medium was replaced with a fresh medium without FBS after seeding the cells for 24 h, and 1 mM 5-ALA (Sigma Aldrich, USA), alone was added. After 4 h of incubation with 5-ALA, the medium of the cells was removed and replaced with the fresh medium containing FBS, and the irradiation protocol was performed. In the irradiation protocol, a diode laser was used with a wavelength of 635 nm ± 3 nm at 1.5, 3, 6, 9, and 12 J/cm^2^ fluence. Therefore, the cells were irradiated and incubated for a further 24 h.

To determine the combination effect of cisplatin-based chemotherapy and 5-ALA-based PDT, two different protocols were performed in MDA-MB-231 cells. In the first protocol, the cells were treated with 1 mM 5-ALA and incubated for 4 h. After incubation, the medium of the cells was replaced with a fresh medium containing FBS, and the cells were irradiated with laser light at fluences of 1.5, 3, 6, 9, and 12 J/cm^2^. After irradiation, 2.5 or 5 µM cisplatin was added into the cells and incubated for 24 h. In the second protocol, the cells were treated with simultaneously 1 mM 5-ALA and 2.5 or 5 µM cisplatin and incubated for 4 h. After incubation, the medium was replaced with fresh medium containing FBS, and the cells were irradiated with a laser with fluences of 1.5, 3, 6, 9, and 12 J/cm^2^. After irradiation, 2.5 or 5 µM cisplatin was added into the cells and the cells were left in the incubator for 24 h. Furthermore, the intracellular protoporphyrin IX (PpIX) level was also observed before irradiation. To determine the intracellular level of PpIX, the cells were treated with 2.5 µM cisplatin and 1 mM 5-ALA combination for 4 h. Then, the cells were captured by the EVOS FL Cell Imaging System (Thermo Fisher Scientific, USA). The amount of intracellular PpIX was analyzed by Image J.

Following treatment with cisplatin or 5-ALA alone and their combinations for 24 h, a WST-1 assay was performed. 10 µl of WST-1 reagent (Biovision, San Francisco, CA, USA) was added in each well and incubated in the dark for 45 min. Following incubation, the cell viability was measured at 450 nm absorbance by an absorbance reader (Allsheng, China).

### Determination of apoptosis by Annexin V

To determine the apoptotic cell death, the cells (1 × 10^5^ cells/well) were seeded in a 12-well plate and incubated at 37 °C with 5% CO_2_. After incubation, the cells treated with cisplatin (2.5 µM) alone, 5-ALA/PDT (1 mM at 6 and 9 J/cm^2^) alone and combination treatment (simultaneously 2.5 µM cisplatin and 1 mM 5-ALA at 6 and 9 J/cm^2^) according to WST-1 analysis. Afterward, the cells were centrifuged at 1500 rpm for 5 min and the supernatant was removed. Following washing with PBS, the Muse® Annexin V & Dead Cell kit (Luminex Corporation, Austin, Texas, USA) was added and incubated for 30 min in the dark. Cell apoptosis was analyzed using the Muse Cell Analyzer (Millipore, Germany).

### Acridine orange staining

For acridine orange staining, the cells ( 5 × 10^5^ cells/well) were seeded in the 12-well plate and incubated for 24 h. After incubation, the cisplatin (2.5 µM), 5-ALA/PDT (1 mM at 6 and 9 J/cm^2^), and combination group (simultaneous 2.5 µM cisplatin and 1 mM 5-ALA at 6 and 9 J/cm^2^) were performed. The cells were fixed with 4% paraformaldehyde and washed with PBS three times. After washing, 100 mg/ml AO (Sigma Aldrich, USA) was added and incubated in the dark for 30 min, and the cells were captured with the EVOS FL Cell Imaging System (Thermo Fisher Scientific, USA).

### Mitochondria staining

To determine changes in the mitochondria of the cells, the cells (5 × 10^5^ cells/well) were seeded in a 12-well plate and the cells were treated as described in the previous section. After incubation for 24 h at 37 °C with 5% CO_2_, the medium was removed from the cells and 100 nM MitoTracker (Thermo Fisher Scientific, USA) solution was added into the cells and incubated for 45 min. Following incubation, the cells were fixed with 4% paraformaldehyde and washed with PBS (Thermo Fisher Scientific, USA). The nucleus of the cells was stained with 4′,6-diamidino-2-phenylindole (DAPI) (Sigma Aldrich, USA) and then imaged using the EVOS FL Cell Imaging System.

### Statistical analysis

SPSS 21.0 (SPSS Inc., Chicago, IL, USA) and GraphPad Prism 6 (La Jolla, USA) were used for statistical analysis in this study. All experiments were replicated three times and the mean standard deviation was calculated. In addition, one-way ANOVA analysis with the post -hoc Tukey test was used for multiple comparisons, *P* < 0.05 was considered significant. The CompuSyn program was used to evaluate the combination efficacy of 5-ALA and cisplatin [17]. As a result of this analysis, those with a combination index (CI) < 1 were considered synergistic, those with CI = 1 were considered as an addictive effect, and those with CI > 1 were considered antagonistic.

## Results

### Determination of intracellular PpIX in MDA-MB-231 and MCF-10A cells

**Fig. 1:**
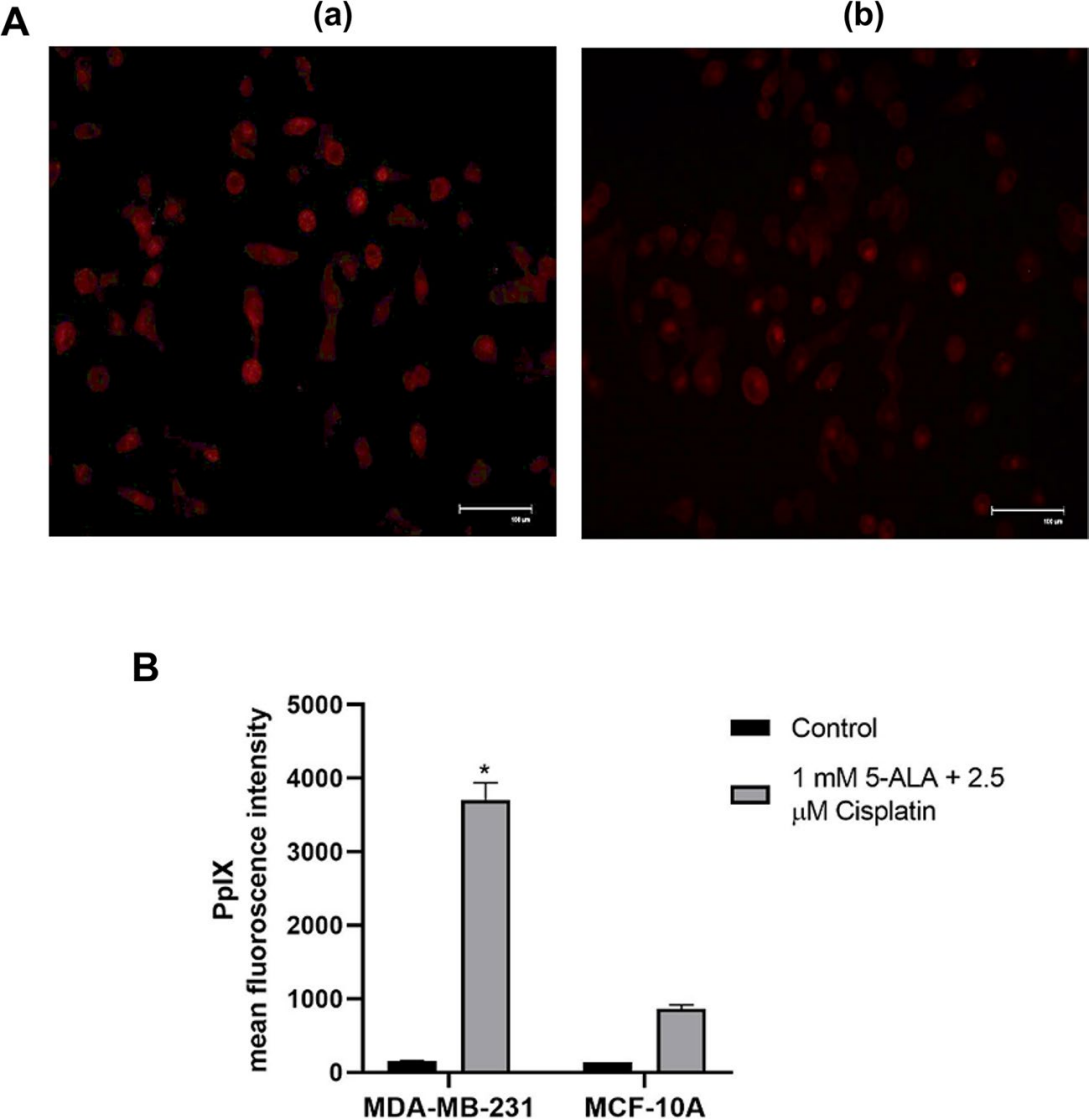
**A** Microscopic images of intracellular PpIX amounts in the cells treated with 2.5 µM cisplatin and 1 mM 5-ALA for 4 h (a) MDA-MB-231 and (b) MCF-10A cells. **B** Statistical comparison of the amount of intracellular fluorescence PpIX in cells compared with the control (*p* < 0.05*)

Intracellular PpIX amount was visualized in 628 ± 40 nm red filter in the cells treated with simultaneously 2.5 µM cisplatin and 1 mM 5-ALA for 4 h. As shown in Fig. 1 there was an increase in the amount of intracellular PpIX accumulated in MDA-MB-231 cells (Fig. 1A (a)) and MCF-10A cells (Fig. 1A (b)) compared to the control group. The mean of fluorescent intracellular PpIX was 3706 ± 233.35 (*p* < 0.05) in the MDA-MB-231 cells group, while the level of PpIX was 864.04 ± 55.20 in MCF-10A cells (Fig. 1B).

### Cytotoxic effects of combination treatment on the cells

**Fig. 2:**
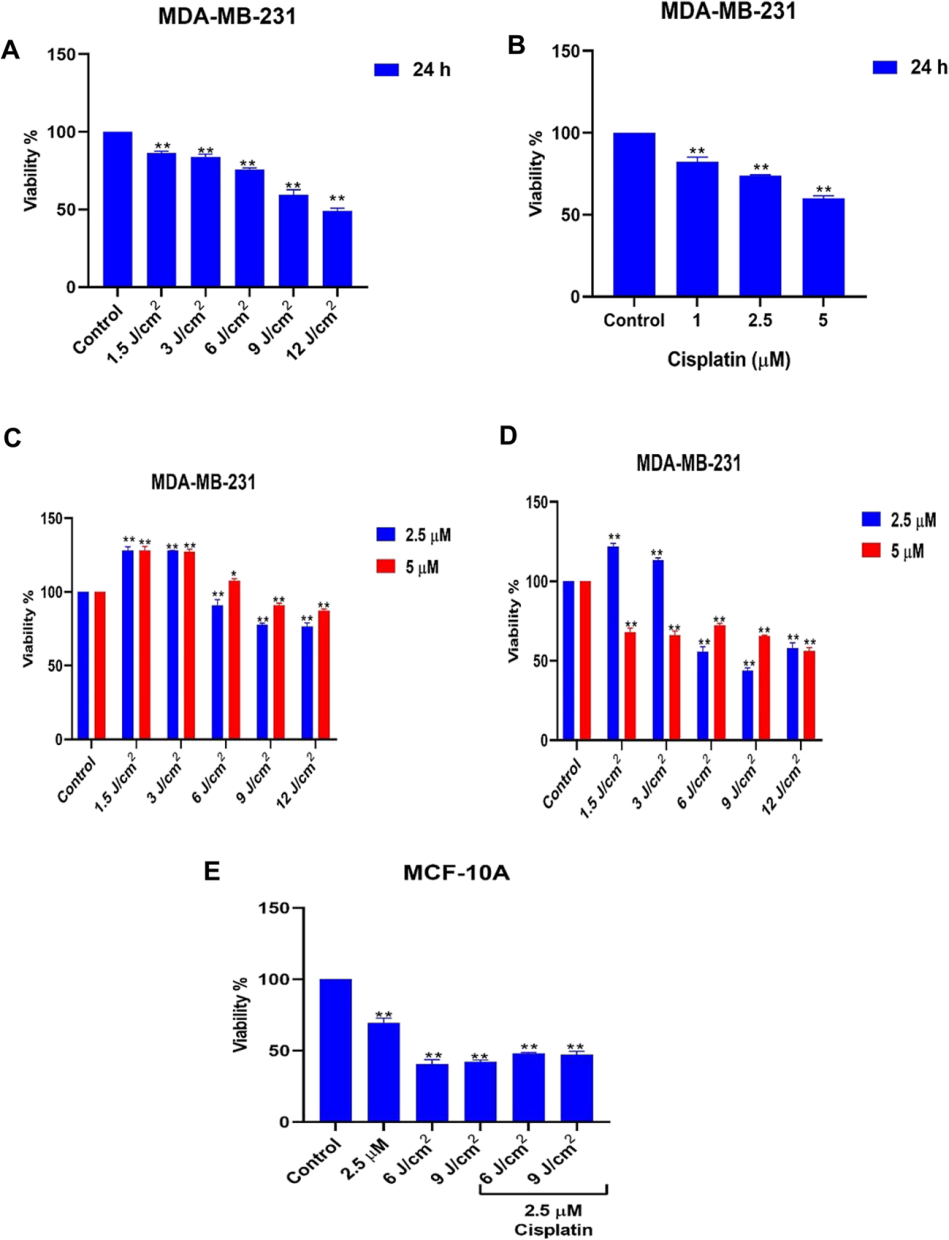
The cytotoxic effects of **A** 5-ALA/PDT alone **B** cisplatin alone in MDA-MB-231 cells **C** The sequential combination and **D** The simultaneous combination of 5-ALA/PDT and cisplatin following irradiation with 1.5 J/cm^2^, 3 J/cm^2^, 6 J/cm^2^, 9 J/cm^2^, and 12 J/cm^2^ **E** The effects of cisplatin, 5-ALA alone or their combination on MCF-10A cells upon irradiation. *p* < 0.05*, *p* < 0.01**)

We first analyzed the cytotoxic effects of 5-ALA/PDT and cisplatin alone on TNBC cells. MDA-MB-231 cells were treated with 5-ALA alone for 4 h. Following irradiation with 1.5, 3, 6, 9 and 12 J/cm^2^, the cell viability reduced to 86.47 ± 1.12%, 83.93 ± 1.80%, 75.97 ± 0.86%, 59.55 ± 3.13% and 48.96 ± 1.79%, respectively for 24 h (*p* < 0.01, Fig. 2A). Our results were consistent with our previous study [12]. MDA-MB-231 cells treated with cisplatin at 1 µM, 2.5 µM and 5 µM concentrations for 24 h, the cell viability was determined as 82.25% ± 2.89%, 73.96% ± 0.48% and 59.96 ± 1.54%, respectively (Fig. 2B, *p* < 0.01**).

In the sequential combination treatment, the cell viability was 128.05 ± 2.60%, 128.12 ± 0.12%, 90.99 ± 3.84%, 77.80 ± 0.99% and 76.51 ± 2.57% at 1.5 J/cm^2^, 3 J/cm^2^, 6 J/cm^2^, 9 J/cm^2^, and 12 J/cm^2^, respectively following treatment with 1 mM 5-ALA and then 2.5 µM cisplatin treatment (Fig. 2C). However, simultaneous treatment was more effective than sequential treatment. Following simultaneous treatment of 2.5 µM cisplatin and 1 mM 5-ALA at the fluences of 1.5 J/cm^2^, 3 J/cm^2^, 6 J/cm^2^, 9 J/cm^2^, and 12 J/cm^2^, the cell viability considerably reduced to 121.89 ± 2.03%, 113.34 ± 1.46%, 55.77 ± 3.00%, 43.83 ± 1.72% and 58.02 ± 3.23%, respectively. On the other hand, 32.07 ± 2.68%, 33.99 ± 2.65%, 27.94 ± 1.33%, 34.40 ± 0.60% and 43.84 ± 2.14%26% reduction was analyzed in cell proliferation following treatment with simultaneous treatment with 5 µM cisplatin and 5-ALA treatment at 1.5 J/cm^2^, 3 J/cm^2^, 6 J/cm^2^, 9 J/cm^2^, and 12 J/cm^2^, respectively (Fig. 2D, *p* < 0.05*). Thus, a simultaneous combination of cisplatin and 5-ALA significantly reduced the viability of TNBC cells and was more effective than monotherapies.

We also statistically determined that 2.5 µM cisplatin and 1 mM 5-ALA treatment upon irradiation with 6 J/cm^2^ and 9 J/cm^2^ had a synergistic effect (CI < 1) in MDA-MB-231 cells, as shown in Table 1. On the other hand, the combination of 5 µM cisplatin with 1 mM 5-ALA following irradiation with all fluences caused an antagonistic effect on TNBC cells.

**Table 1:** CI values for the simultaneous combination treatment of cisplatin and 5-ALA at different fluence rates of irradiation. The CI values were determined to analyze the interaction between cisplatin and 5-ALA treatment in TNBC cells

Cisplatin	2.5 µM	5 µM
CI at 1.5 J/cm^2^	762.92	1.68
CI at 3 J/cm^2^	957.64	1.74
CI at 6 J/cm^2^	0.93	2.97
CI at 9 J/cm^2^	0.71	2.52
CI at 12 J/cm^2^	1.62	1.91

Additionally, we analyzed the toxicity of a simultaneous combination cisplatin and 5-ALA on MCF-10A control cells. The viability of MCF-10A cells treated with 1 mM 5-ALA reduced to 40.54 ± 3.19% and 42.22 ± 1.23% at 6 J/cm^2^ and 9 J/cm^2^, whereas the cell viability was 48.10 ± 0.55% and 47.25 ± 2.52%, at 6 J/cm^2^ and 9 J/cm^2^, respectively upon treatment with 2.5 µM cisplatin and 1 mM 5-ALA simultaneously (Fig. 2E, *p* < 0.01**). Furthermore, 2.5 µM cisplatin alone treatment reduced the cell viability to 68.64 ± 3.15% in the MCF-10A cell viability. Therefore, simultaneous treatment reduced the toxicity of 5-ALA alone in MCF-10A cells.

### Apoptotic effects of combination treatment on the cells

**Fig. 3:**
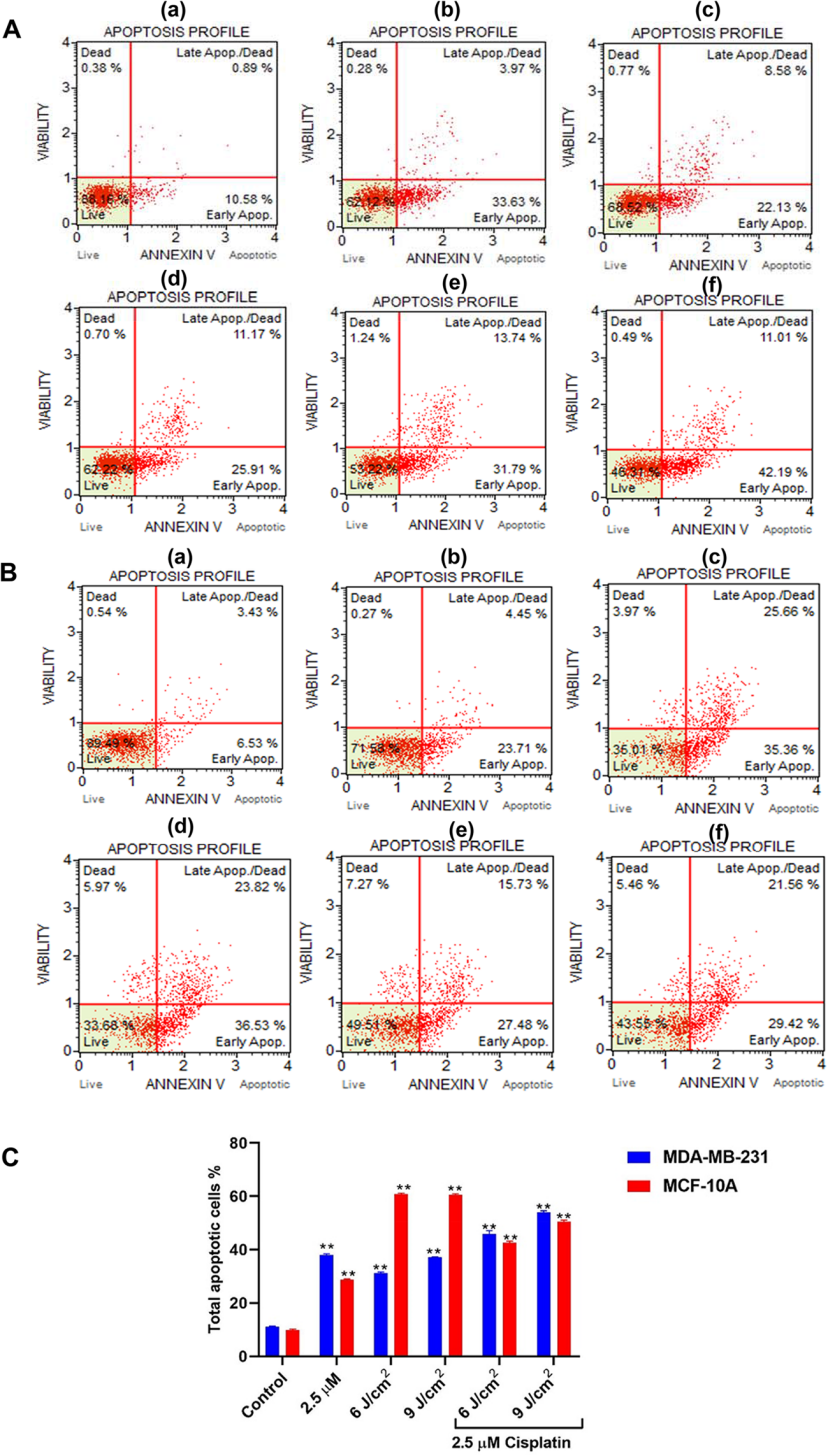
**A** The histograms of Annexin V analysis in **A** MDA-MB-231 and **B** MCF-10A cells. **a** control group, **b** 2.5 µM cisplatin monotherapy, 1 mM 5-ALA treatment upon irradiation with **c** 6 J/cm^2^, **d** 9 J/cm^2^ irradiation, the cells treated with 2.5 µM cisplatin with 1 mM 5-ALA at **e** 6 J/cm^2^ and **f** 9 J/cm^2^. **C** Statistical comparison of total apoptotic cell death in MDA-MB-231 and MCF-10A cells after cisplatin, 5-ALA/PDT, and simultaneous combination treatments (*p* < 0.01**)

In MDA-MB-231 cells, the percentage of early apoptotic cells increased to 46.78% and 53.6% in simultaneous combination treatment of 2.5 µM cisplatin and 5-ALA/PDT at 6 J/cm^2^ and 9 J/cm^2^, respectively compared to 2.5 µM cisplatin monotherapy (37.88%) and 5-ALA/PDT monotherapy (6 J/cm^2^: 31.48% and 9 J/cm^2^: 37.78%). Thus, especially more early apoptotic cell death was determined in the TNBC cells upon combination treatment compared to monotherapies (Fig. 3, *p* < 0.01**).

While the total percentage of apoptotic death in MCF-10A cells was 28.42% upon treatment with 2.5 µM cisplatin monotherapy, the rate of death was analyzed as 64.99% and 66.32% after irradiation with the fluences of 6 J/cm^2^ and 9 J/cm^2^ in 5-ALA-treated cells, respectively. In the combination treatment, the total percentage of apoptotic cells increased to 50.49% and 56.45% at 6 J/cm^2^ and 9 J/cm^2^, respectively (Fig. 3, *p* < 0.01**).

### Evaluation of morphological changes caused by 5-ALA-Based PDT and cisplatin combination treatment in the cells

In MDA-MB-231 and MCF-10A cells, chromatin condensation, DNA and nuclear fragmentation were observed in the cells treated with cisplatin, 5-ALA/PDT and combination treatment compared to the control group.

**Fig. 4:**
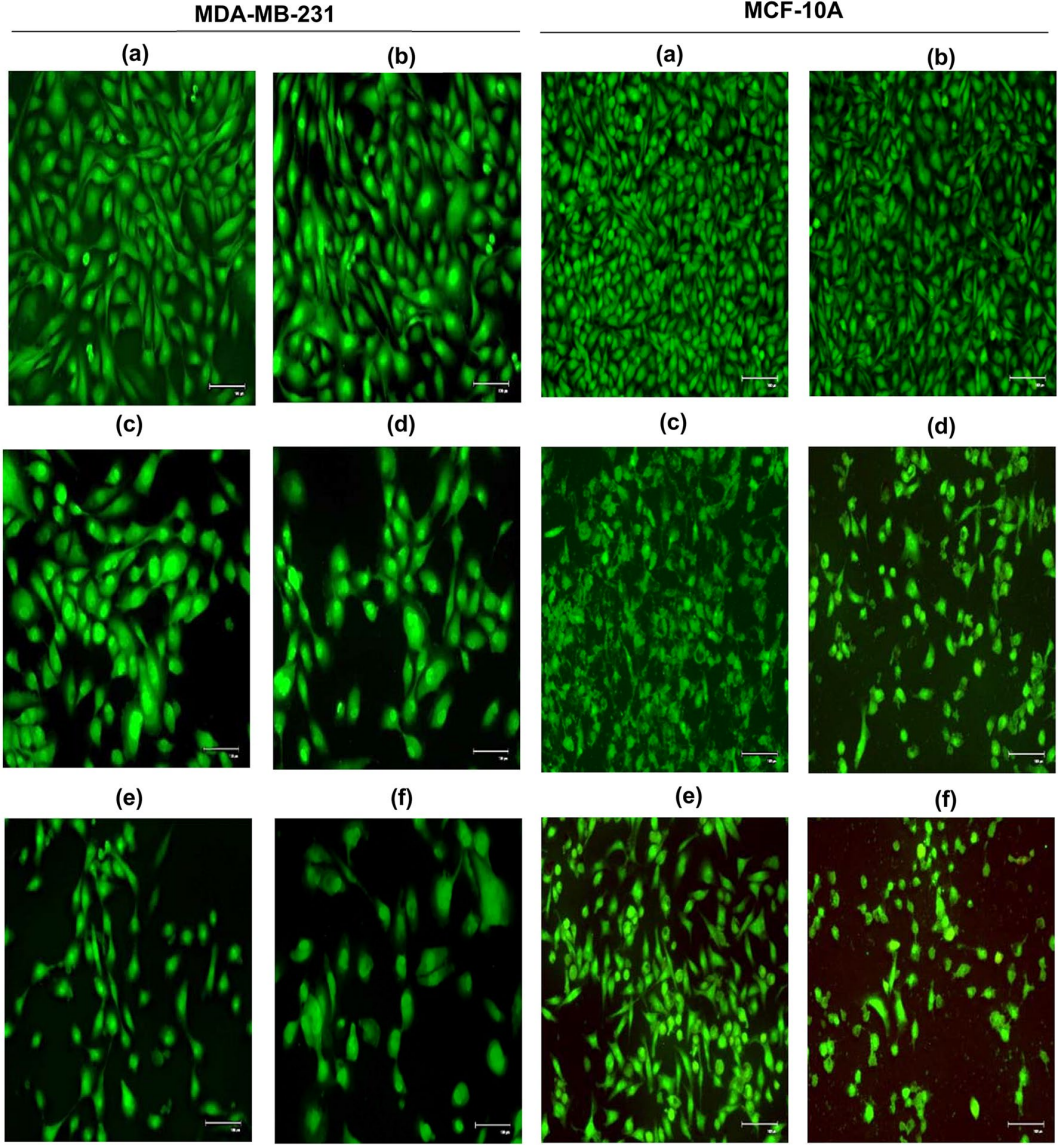
Morphological changes in MDA-MB-231 and MCF-10A cells treated with cisplatin, 5-ALA/PDT monotherapies and combination treatment were observed by AO staining. **a** control group, **b** 2.5 µM cisplatin monotherapy, 1 mM 5-ALA treatment upon irradiation with **c** 6 J/cm^2^, **d** 9 J/cm^2^ irradiation, the cells treated with 2.5 µM cisplatin with 1 mM 5-ALA at **e** 6 J/cm^2^ and **f** 9 J/cm^2^

After combination treatment of 2.5 µM cisplatin and 1 mM 5-ALA/PDT in MDA-MB-231 cells, more distinct nuclear fragmentation and rupture of cell–cell connections were observed at especially 9 J/cm^2^. On the other hand, combination treatment resulted in less damage in MCF-10A cells than 5-ALA/PDT monotherapy (Fig. 4). However, cellular swelling was observed following treatment with 5-ALA/PDT alone and in combination with cisplatin in MDA-MB-231 and MCF-10A cells due to possible necrotic cell death.

### Mitochondrial changes caused by 5-ALA-Based PDT and cisplatin combination treatment in the cells

**Fig. 5:**
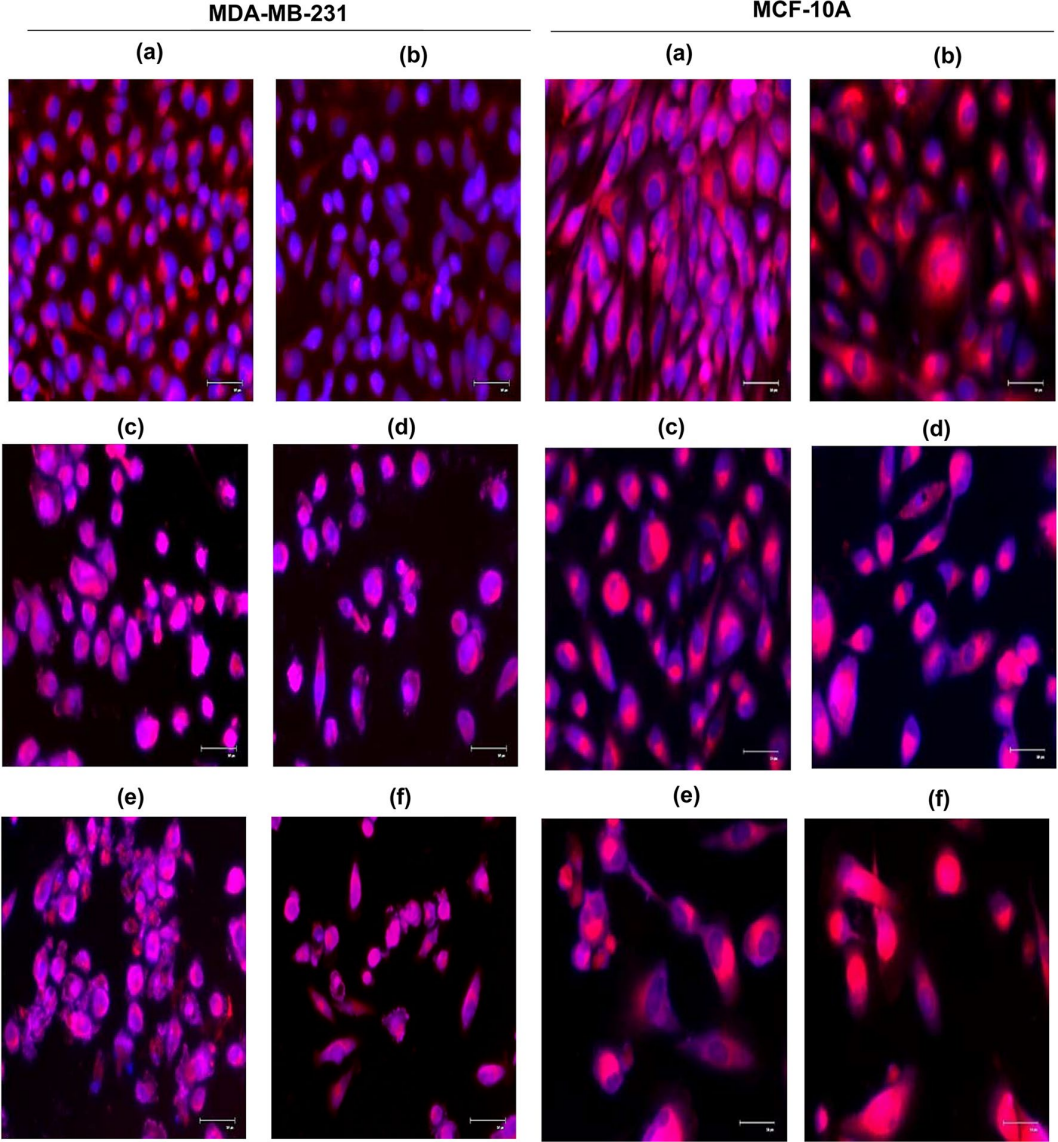
To verify apoptosis, mitochondrial damages were analyzed by MitoTracker in MDA-MB-231 and MCF-10A cells upon cisplatin and 5-ALA/PDT monotherapies and combination treatment. **a** control group, **b** 2.5 µM cisplatin monotherapy, 1 mM 5-ALA treatment upon irradiation with **c** 6 J/cm^2^, **d** 9 J/cm^2^ irradiation, the cells treated with 2.5 µM cisplatin with 1 mM 5-ALA at **e** 6 J/cm^2^ and **f** 9 J/cm^2^

In MDA-MB-231 and MCF-10A cells, a decrease in mitochondrial fluorescence intensities was observed following cisplatin monotherapy compared to the control group. In contrast, increased fluorescence intensities in the mitochondria were detected in cells treated with 5-ALA/PDT monotherapy and combination treatment consistent with the increased energy densities. Furthermore, many fragmented mitochondria in TNBC cells were observed, especially in the combination treatment group (Fig. 5). On the other hand, more fragmented mitochondria were detected in 5-ALA/PDT monotherapy compared to the combination treatment in MCF-10A cells (Fig. 5).

## Discussion

Herein, our findings indicated that the combination treatment of cisplatin and 5-ALA/PDT following irradiation at 6 J/cm^2^ and 9 J/cm^2^ showed more therapeutic potency than cisplatin and 5-ALA/PDT monotherapies in TNBC cells with less toxicity in control cells. Furthermore, this combination induced apoptotic cell death and nuclear damage in TNBC cells.

Certain chemotherapeutic drugs, including taxanes, anthracyclines and platinum, are used as current treatment options in TNBC. However, the development of drug resistance and the adverse side effects of these drugs, including nephrotoxicity, ototoxicity and hepatotoxicity, remarkably limit their effectiveness [18–20]. Therefore, developing combination treatment options targeting specific molecules could be beneficial in treating TNBC [21, 22].

PDT is a new treatment method causing different types of death in cancer cells through the production of ROS due to the activation of PS via the absorption of light energy at a specific wavelength. When PS is activated by light of a specific wavelength, it interacts with oxygen molecules in the tissue and initiates chemical reactions, producing toxic products and causing the death of targeted cancer cells [23–25]. Although PDT has some advantages, such as non-invasiveness, short-time application, and reproducibility, some side effects, selectivity and penetration problems, and oxygen concentration reduce the therapeutic efficacy of PDT in cancer treatment [26–28]. In this context, we focused on combining chemotherapy and 5-ALA-based PDT in TNBC treatment to overcome these problems.

5-ALA, a natural amino acid required for heme synthesis, is a common type of PS used in treating PDT. In tumor tissues, PpIX accumulates in cells following the changes in the activity of the heme synthesis pathway of 5-ALA. After the fluorescent molecule PpIX accumulates in cancer cells, it causes ROS production, damage to mitochondrial and cytoplasmic proteins, and death in cancer cells when stimulated with light [29]. In the literature, 5-ALA-based PDT exerts a significant anticancer in different cancer cell lines due to increased intracellular PpIX [10–13]. In our previous study, 5-ALA treatment resulted in a considerable decrease in the viability of MCF-7 and MDA-MB-231 cells upon irradiation at 1.5, 3, 6, 9 and 12 J/cm^2^. Additionally, MDA-MB-231 cells are more sensitive to 5-ALA/PDT at 9 and 12 J/cm^2^ [12]. In this study, the combination treatment of 5-ALA/PDT with cisplatin in MDA-MB-231 cells caused a significant increase in intracellular PpIX in the cells compared with the control. Therefore, the higher intracellular accumulation of PpIX may associated with higher therapeutic synergistic efficacy of cisplatin and 5-ALA/PDT combination therapy compared to 5-ALA/PDT monotherapy.

In the literature, the combination effects of different chemotherapy drugs and PS-based PDT have been investigated in various cancer types [30–34]. In the study of Javani Jouni et al. (2022), the combination effects of cisplatin and methylene blue-based PDT following irradiation with a laser of 630 nm at the fluence of 4 J/cm^2^ on cervical cancer cells (A2780 and A2780-CP) cells are assessed. In this study, combination therapy significantly reduces cell viability compared to monotherapies by increasing concentrations of cisplatin [30]. Zakaria et al. (2014) state that doxorubicin and 5-ALA treatment causes a more significant decrease in the viability of MCF-7 cells compared to monotherapies [31]. Furthermore, some sequential treatment protocols for the combination of chemotherapy and PDT are more effective in some cancer types. Chul Ahn et al. (2014) note that the sequential treatment of cisplatin and 5-ALA based PDT at 6 J/cm^2^ combination exerts more significant anticancer activity in human head and neck squamous carcinoma cells [32]. In a study conducted by Sadeghloo et al. (2020), MDA-MB-231 cells are treated with 15 µg/ml methylene blue for 2 h and irradiated with 660 nm light with the fluence of 3 J/cm^2^. Then, the cells are incubated with different concentrations of doxorubicin. Additionally, the cells are firstly treated with doxorubicin and then methylene blue for different incubation times. According to the obtained data, the sequential treatment of methylene blue and then doxorubicin is the most effective combination treatment option in TNBC cells [33]. Therefore, the identification of treatment and irradiation protocol (sequential or simultaneous) for each chemotherapy drug and PS plays a crucial role in effective treatment. In this context, we, for the first time, assessed the therapeutic effects of a combination of 5-ALA/PDT with cisplatin on TNBC cells and control cells. Our findings demonstrated that the simultaneous combination treatment of cisplatin and 5-ALA/PDT after irradiation with the fluence of 6 and 9 J/cm^2^ was more effective than cisplatin or 5-ALA/PDT monotherapy or the sequential treatment protocol in TNBC cells and caused apoptotic cell death via nuclear and mitochondrial damages. However, higher concentrations (5 µM) of cisplatin with 5-ALA combination after irradiation exerted antagonistic effects on TNBC cells. Additionally, the sequential treatment was not effective in TNBC cells. In the literature, combining different chemotherapeutic drugs with other PS exhibits synergistic or antagonistic effects depending on the concentrations, exposure times, treatment sequence, and cancer type [35, 36]. In fact, the interaction between PDT and chemotherapy drugs could rely on the generation of singlet oxygen amount. Therefore, some of the singlet oxygen generated from 5-ALA could be hindered by cisplatin and this treatment can lead to antagonism in TNBC cells [36]. Additionally, 5-ALA/PDT at especially 9 J/cm^2^ treatment induced significant late apoptotic cells. This phototoxicity could be associated with a higher amount of singlet oxygen. Therefore, the underlying molecular mechanisms behind the efficacy of cisplatin and 5-ALA simultaneous combination in TNBC cells should be further elucidated to obtain maximum synergism and minimum antagonism between 5-ALA/PDT and cisplatin with minimum toxicity in control cells.

## Conclusion

In conclusion, our results suggest that cisplatin and 5-ALA-based PDT combination upon irradiation with 6 and 9 J/cm^2^ exert synergistic effects on TNBC cells and induce apoptotic cell death through mitochondrial damages. Additionally, the simultaneous treatment protocol is more effective than the sequential treatment. As a result of the combination treatment, the effective concentration of cisplatin and the fluences for the stimulation of 5-ALA can be reduced, and the side effects of 5-ALA following irradiation can be prevented. Conversely, cisplatin and 5-ALA/PDT combination treatment had toxic effects on MCF-10A cells. Therefore, nanoparticle drug delivery systems could be developed to reduce the toxicity of cisplatin and 5-ALA and increase the target tissue's selectivity. Additionally, further combination treatment strategies should be investigated in TNBC cells.
